# Gastrointestinal endoscopy for physiological assessment in functional gastrointestinal disorders: a perspective

**DOI:** 10.3389/fmed.2026.1878949

**Published:** 2026-07-10

**Authors:** Jing-rong Dong, Shan-shan Guo, Bin Hu, Wen-shuang Zou

**Affiliations:** 1 Department of Gastrointestinal Endoscopy, The Affiliated Hospital of Changchun University of Chinese Medicine, Changchun, China; 2 Finance Office, The Affiliated Hospital of Changchun University of Chinese Medicine, Changchun, China; 3 Discipline Inspection Commission Office, The Affiliated Hospital of Changchun University of Chinese Medicine, Changchun, China; 4 Department of Gastroenterology, The Affiliated Hospital of Changchun University of Chinese Medicine, Changchun, China

**Keywords:** disorders of gut–brain interaction, endoscopic imaging, functional gastrointestinal disorders, gastrointestinal endoscopy, optical biopsy, physiological assessment, precision phenotyping

## Abstract

The diagnosis of functional gastrointestinal disorders, now conceptualized as disorders of gut–brain interaction (DGBI), has long relied on symptom-based criteria. Gastrointestinal endoscopy, meanwhile, has been largely confined to the exclusion of organic pathology. This exclusionary logic has led to a systematic underestimation of the diagnostic value embedded in endoscopically normal findings. Here we propose a fundamental repositioning of the endoscope from a static morphological exclusion tool into a dynamic physiological assessment platform. By integrating real-time intraluminal functional imaging, optical biopsy of mucosal microarchitecture, neuro-immune fluorescence molecular imaging, and intraluminal environmental monitoring, this platform enables the *in situ* capture of key pathophysiological parameters—including gastrointestinal sensorimotor function, mucosal barrier integrity, and immune activation status. Establishing such a “functional endoscopy” assessment framework may help bridge the traditional divide between structural and functional disease. It could also facilitate future precision endoscopic phenotyping of DGBI based on underlying pathophysiological mechanisms.

## 1 Introduction

### 1.1 Ceiling of the exclusionary diagnostic paradigm

Under the Rome IV framework, gastrointestinal endoscopy is assigned a narrowly negative role: it is invoked only to exclude organic pathology when alarm features are present (1, 2). This paradigm rests on a simple binary logic—if the mucosa appears structurally intact, symptoms are attributed to a functional disorder (1, 2). Clinical practice, however, repeatedly exposes the weakness of this reasoning. A substantial proportion of endoscopic examinations yield negative findings, yet patients continue to suffer from persistent and distressing symptoms (3). The resulting disconnect between diagnostic output and lived illness experience produces tangible harm: diagnostic delays are prolonged, patient anxiety is amplified, and healthcare resources are inefficiently consumed (3, 4). We contend that this exclusionary model has reached its ceiling in clinical utility and cannot advance without a fundamental conceptual shift.

### 1.2 Imperative for a paradigmatic shift in perspective

The pathophysiological understanding of functional gastrointestinal disorders has long moved beyond a unidimensional focus on dysmotility (4, 5). A multidimensional model now prevails, recognizing that disordered motility, visceral hypersensitivity, impaired mucosal barrier function, low-grade immune activation, and gut microbiota dysbiosis do not operate in isolation (4–6). Rather, they form a dynamically interacting network that collectively constitutes the biological substrate of symptoms (5, 6). This complex architecture cannot be captured by the reductive equation that equates structural normality with functional integrity. We therefore advance a core thesis: the field requires a fundamental shift in perspective. In concrete terms, the endoscope should be redefined as an active intraluminal physiological probing platform, rather than a passive recorder of macroscopic morphology (7, 8). Within the procedural context of endoscopy itself, this platform would enable the real-time, *in situ* capture of motility patterns, assessment of barrier competence, and quantification of sensory thresholds (8). Only through such a paradigm shift—from morphological exclusion to functional capture—can the diagnostic impasse built around the concept of “normal mucosa” be dismantled, opening a new pathway toward precision diagnostics in functional gastrointestinal disorders.

### 1.3 Positioning functional endoscopy within existing endoscopic paradigms

The concept of functional endoscopy should be distinguished from existing technologies such as physiological endoscopy, functional lumen imaging probe panometry, high-resolution manometry-assisted endoscopy, or individual endo-functional assessment techniques (9, 10). These existing approaches primarily evaluate specific physiological domains, most commonly motility or luminal distensibility (9, 11). In contrast, functional endoscopy is proposed as an overarching conceptual framework rather than as a single technology. Its defining characteristic is the integration of multiple physiological dimensions within a unified endoscopic platform (10, 12). These dimensions include barrier integrity, neuroimmune activation, intraluminal environmental dynamics, and gut–brain interaction testing (10, 12, 13). Therefore, the novelty of functional endoscopy lies in its multidimensional, mechanism-oriented phenotyping strategy for disorders of gut–brain interaction (DGBI), not in any individual assessment modality.

## 2 Imaging bottleneck in the pathophysiology of functional gastrointestinal disorders

### 2.1 Physiological blind spot of conventional endoscopy

White-light endoscopy, the most widely used luminal imaging modality in clinical practice, is fundamentally designed for macroscopic morphological assessment (14). It illuminates the tissue surface with broadband light and reconstructs mucosal contours, color, and texture from the reflected signals. This operating principle inherently limits its informational scope. Core pathophysiological processes in functional gastrointestinal disorders—such as dynamic changes in mucosal barrier permeability, microcirculatory dysregulation within the submucosa, activation of subepithelial nerve terminals, and low-grade immune cell infiltration—all occur beyond the spatial resolution and molecular information capacity of white-light endoscopy (4, 5, 15). The biological events that drive symptom generation thus remain categorically invisible to conventional visualization (4, 5, 15).

### 2.2 Technological bridges across the divide

To overcome this bottleneck, several emerging platforms that combine advanced optics, physiological sensing, and molecular labeling are being integrated into the endoscopic workflow (11, 14). Together, they form the technological bridge for the requisite paradigm shift. These technologies fall into three categories.

The first is the deepening of optical biopsy. Techniques such as confocal laser endomicroscopy and endocytoscopy enable real-time, subcellular-resolution imaging of the mucosa *in situ* (14, 16). They render microstructural markers of barrier impairment—including epithelial gap dilatation and cell shedding—directly visible, advancing endoscopic diagnosis from macroscopic morphology to the microscopic level (11, 16, 17).

The second is the integration of functional sensing. Endoluminal impedance–pressure catheters and high-resolution manometry can precisely quantify intraluminal pressure gradients and bolus transit characteristics in the esophagus and anorectum (18). When coupled with endoscopic platforms, these sensing modalities allow simultaneous acquisition of regional motility parameters alongside morphological observation, providing direct physiological evidence for symptoms attributable to dysmotility (19, 20).

The third is the targeting capacity of molecular imaging. The intravenous or topical administration of fluorescently labeled probes enables *in vivo* molecular imaging of specific receptors, cytokines, or adhesion molecules (20, 21). This approach holds the potential to reveal the spatial distribution and dynamic evolution of molecular events such as immune activation and neurogenic inflammation, thereby equipping the endoscope with molecular recognition capabilities (20, 21).

These three technological trajectories do not evolve in isolation. Rather, they converge upon a unified vision: transforming the endoscope from a passive descriptor of anatomical form into an active intraluminal platform capable of capturing the multidimensional pathophysiological signatures of functional gastrointestinal disorders (20–22).

## 3 Four physiological dimensions for constructing “functional endoscopy”

**Figure 1:**
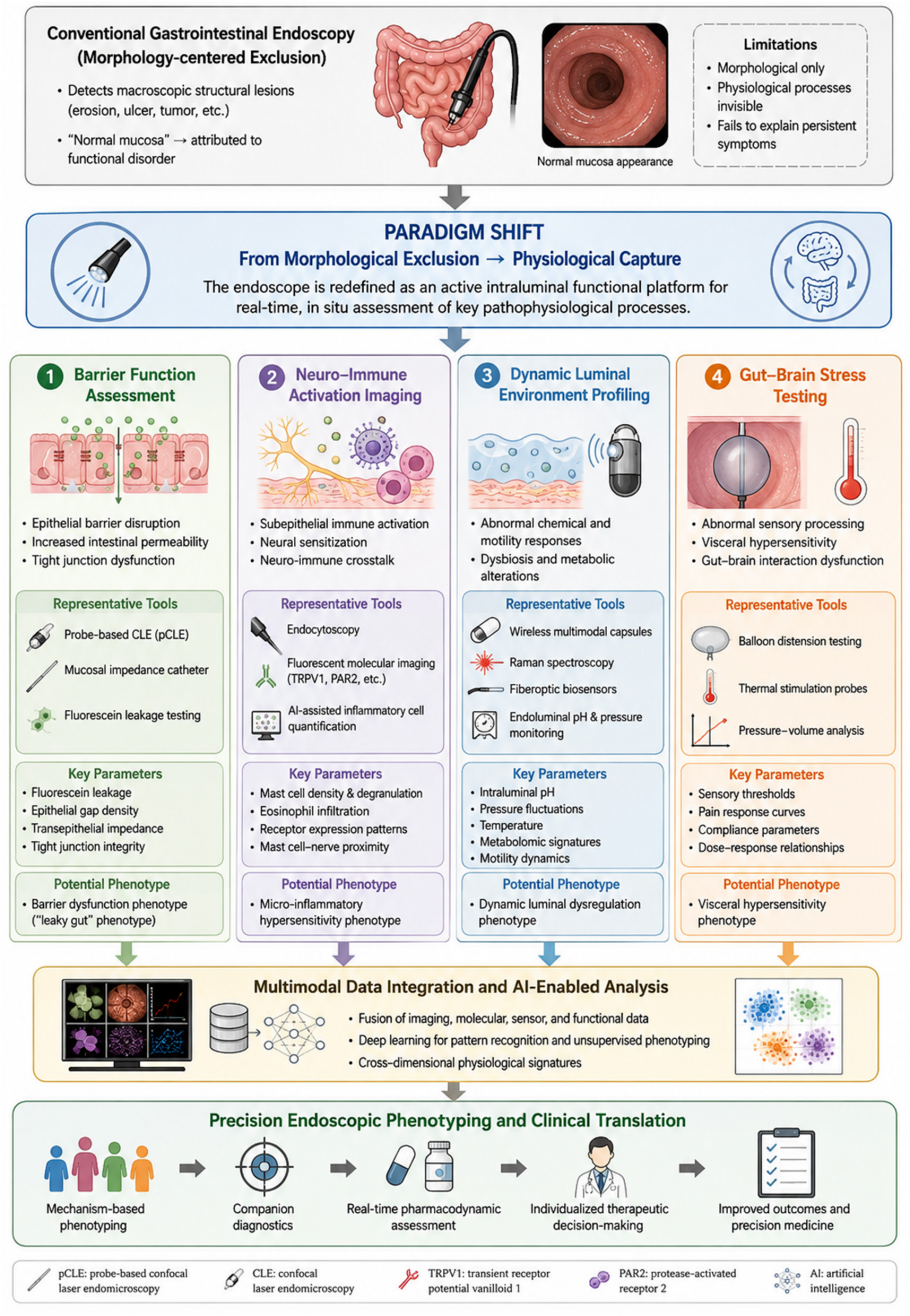
Conceptual framework of functional endoscopy for physiological phenotyping in disorders of gut-brain interaction.

The overall conceptual framework of functional endoscopy is summarized in Figure 1. It departs from a technology-centered classification and instead adopts a physiological system-centered approach, establishing four evaluative dimensions for functional endoscopy. Importantly, not all dimensions are equally mature. Some technologies, such as mucosal impedance assessment and functional lumen imaging probe panometry, are already clinically available, whereas molecular neuro-immune imaging remains largely experimental. Therefore, we envision a staged translational pathway in which currently available physiological tools serve as the foundation for progressively more advanced functional endoscopic platforms (Table 1).

**Table 1:** Translational maturity of functional endoscopy components.

Dimension	Current clinical availability	Near-term translation	Exploratory future applications
Barrier function	Mucosal impedance	CLE permeability imaging	Real-time permeability mapping
Neuro-immune activation	Histology	AI-assisted endocytoscopy	Molecular fluorescence imaging
Intraluminal profiling	Manometry/FLIP	Integrated multimodal sensing	Real-time metabolomics
Gut-brain testing	Balloon distension	Endoscopic sensory testing	Closed-loop neurophysiology

CLE, Confocal Laser Endomicroscopy; FLIP, Functional Lumen Imaging Probe; AI, Artificial Intelligence.

### 3.1 Real-time endoscopic assessment of mucosal barrier function

The conceptual innovation lies in shifting from observing morphology to measuring leakage. Conventional endoscopy detects only macroscopic mucosal defects and remains blind to subclinical barrier impairment (23, 24). Technologically, the fluorescein leakage test under probe-based confocal laser endomicroscopy enables real-time visualization of fluorescent tracer extravasation across dilated epithelial gaps, thereby directly quantifying mucosal permeability (23). Concurrently, novel balloon-tipped electrode catheters, introduced through the biopsy channel, are making endoscopic mucosal impedance measurement feasible—detecting alterations in transepithelial electrical resistance as a reflection of tight junction integrity (25). Clinically, this dimension allows direct confirmation of duodenal barrier disruption in patients with functional dyspepsia and diarrhea-predominant irritable bowel syndrome (26, 27). By providing morphological evidence of a “leaky gut” at the level of epithelial cell junctions, it offers a definitive pathophysiological substrate for postprandial bloating and visceral pain (27).

### 3.2 *In situ* imaging of sensory neural and immune activation

The conceptual innovation lies in shifting from inspecting for erosions to probing for hypersensitivity. In functional gastrointestinal disorders, the biological basis of visceral hypersensitivity is often concealed beneath an apparently normal mucosal surface, manifesting as low-grade neuro-immune dysregulation (28). Technologically, endocytoscopy combined with artificial intelligence algorithms allows quantitative assessment of eosinophil and mast cell density and degranulation within the mucosa (29, 30). Fluorescently labeled probes targeting the transient receptor potential vanilloid 1 or protease-activated receptor 2 enable *in vivo* labeling of key nodes in nociceptive signaling pathways (31, 32). Clinically, this dimension permits the identification of an endoscopic micro-inflammatory phenotype driving visceral hypersensitivity within non-erythematous, non-erosive mucosa (33). Direct visualization of mast cell–nerve fiber co-localization, for example, transforms “occult sensitivity” into a visible diagnostic entity (33).

### 3.3 Dynamic profiling of the intraluminal physiological environment

The conceptual innovation lies in shifting from a static snapshot to a dynamic profile. Conventional endoscopy yields isolated, instantaneous images that fail to capture the spatiotemporal evolution of gastrointestinal physiological parameters (34). Technologically, wireless multimodal capsules deployed endoscopically can synchronously record intraluminal pH, pressure, and temperature (35). Simultaneously, Raman spectroscopy and fiberoptic biosensors enable *in situ* metabolomic analysis of luminal fluid (36). Clinically, this dimension makes it possible to capture—in real time during the endoscopic procedure—abnormal motor and chemosensory responses of the duodenum to acid or lipid perfusion (37). It renders functional abnormalities directly recordable as dynamic video sequences, rather than relying on retrospective inference.

### 3.4 Interventional stress testing of gut–brain interactions under endoscopy

The conceptual innovation lies in shifting from resting observation to provocative testing. The episodic nature of symptom occurrence often precludes the capture of critical pathophysiological states during resting-state endoscopy alone (38, 39). Technologically, endoscopic balloon distension can generate standardized pressure–volume curves to assess visceral hypersensitivity, while thermal stimulation probes enable precise determination of rectal sensory thresholds (40). Clinically, this dimension translates the highly subjective pain scores enshrined in the Rome criteria into objective, reproducible neurophysiological dose–response relationships (38, 40, 41). It thereby compensates for the inherent specificity limitations of symptom-clustering models and provides an experimental medicine dimension for diagnosing DGBI. This framework repositions the endoscope as an experimental medicine platform targeting the human gut–brain axis *in vivo*. The four physiological dimensions, representative technologies, measurable parameters, and corresponding clinical implications are summarized in Table 2.

**Table 2:** Four physiological dimensions of functional endoscopy and their corresponding technologies and clinical implications.

Physiological dimension	Core pathophysiological target	Representative technologies	Key measurable parameters	Potential endoscopic phenotype	Clinical significance
Real-time assessment of mucosal barrier function	Epithelial barrier disruption and increased intestinal permeability	Probe-based CLE; mucosal impedance catheter; fluorescein leakage testing	Fluorescein extravasation; epithelial gap density; transepithelial impedance values; tight junction integrity	Barrier dysfunction phenotype (“leaky gut” phenotype)	Provides objective evidence for impaired epithelial integrity in functional dyspepsia and IBS-D; links postprandial bloating and visceral pain to measurable barrier abnormalities
*In situ* imaging of sensory neural and immune activation	Neuro-immune dysregulation and visceral hypersensitivity	Endocytoscopy; AI-assisted inflammatory cell quantification; fluorescent molecular probes targeting TRPV1 and PAR2	Mast cell density and degranulation; eosinophil infiltration; receptor expression patterns; mast cell–nerve proximity	Micro-inflammatory hypersensitivity phenotype	Enables visualization of occult neuro-immune activation beneath macroscopically normal mucosa; supports mechanism-based identification of visceral hypersensitivity
Dynamic profiling of the intraluminal physiological environment	Abnormal luminal chemical and motility responses	Wireless multimodal capsules; Raman spectroscopy; fiberoptic biosensors; endoluminal pressure and pH monitoring	Intraluminal pH; pressure fluctuations; temperature; metabolomic spectral signatures; motility dynamics	Dynamic luminal dysregulation phenotype	Captures real-time physiological responses to acid, lipid, or nutrient stimulation; transforms functional abnormalities into directly recordable physiological events
Interventional stress testing of gut–brain interactions	Abnormal sensory processing and gut–brain interaction dysfunction	Balloon distension testing; thermal stimulation probes; pressure–volume curve analysis	Sensory thresholds; pain response curves; compliance parameters; neurophysiological dose–response relationships	Visceral hypersensitivity phenotype	Converts subjective symptom perception into reproducible physiological measurements; improves mechanistic phenotyping of disorders of gut–brain interaction
AI–assisted multimodal integration	Cross-dimensional physiological pattern recognition	Deep learning algorithms; multimodal data fusion platforms; AI-assisted endoscopic analytics	Integrated physiological signatures; predictive phenotypic clustering; multidimensional biomarker patterns	AI-derived precision phenotype	Facilitates unsupervised subtype discovery and individualized prediction; supports mechanism-driven classification and precision medicine
Intrinsic therapeutic coupling and companion diagnostics	Real-time assessment of therapeutic responsiveness	Endoscopic local drug perfusion; real-time molecular imaging; functional monitoring under pharmacologic intervention	Dynamic changes in barrier function; receptor activity modulation; sensory response alterations	Therapy-responsive endoscopic phenotype	Enables *in vivo* drug sensitivity testing during endoscopy; shifts management from empirical therapy toward individualized precision intervention

AI, artificial intelligence; CLE, confocal laser endomicroscopy; IBS-D, diarrhea-predominant irritable bowel syndrome; TRPV1, transient receptor potential vanilloid 1; PAR2, protease-activated receptor 2.

## 4 Challenges and critical reflections: distance from laboratory to endoscopy suite

Although the technological blueprint outlined above delineates a promising horizon for functional endoscopy, translating it from proof-of-concept to routine clinical practice should confront several unavoidable realities. The major translational barriers and future directions for functional endoscopy are summarized in Table 3.

**Table 3:** Challenges and translational barriers in the clinical implementation of functional endoscopy.

Challenge domain	Specific issue	Potential clinical impact	Proposed solutions/future directions
Procedure time and workflow burden	Integration of physiological testing modules may substantially prolong endoscopic procedures	Reduced procedural efficiency; increased operator fatigue; decreased endoscopy unit throughput	Develop modular and selectively deployable assessment protocols; prioritize targeted physiological testing based on clinical indications
Economic cost and resource allocation	Advanced imaging systems, biosensors, molecular probes, and AI platforms require significant financial investment	Increased healthcare expenditure; limited accessibility in resource-constrained centers	Establish cost-effectiveness models; improve diagnostic yield through targeted biopsies and precision phenotyping; promote scalable platform development
Lack of standardized interpretative criteria	Absence of unified thresholds for impedance values, fluorescein leakage, inflammatory cell counts, and sensory testing	Interobserver variability; inconsistent diagnosis; limited reproducibility across centers	Develop multicenter prospective cohorts; establish reference ranges and consensus reporting standards similar to the Chicago Classification
Risk of technology-driven overdiagnosis	Physiological deviations without validated clinical significance may be pathologized prematurely	Unnecessary interventions; patient anxiety; overtreatment	Emphasize clinical utility validation; integrate symptom correlation and longitudinal outcome assessment before routine implementation
Sedation-related physiological interference	Deep sedation may alter motility, sensory perception, and neuromuscular responses	Reduced physiological authenticity of functional measurements	Optimize sedation protocols; develop minimally sedated or conscious-state assessment strategies for selected procedures
Patient tolerance and acceptability	Balloon distension, perfusion testing, and prolonged procedures may increase discomfort and psychological stress	Reduced patient compliance; lower acceptance of repeated testing	Use patient-centered communication; implement shorter modular testing strategies; improve procedural comfort technologies
Technical complexity and operator dependency	Functional endoscopy requires multidisciplinary expertise in imaging, physiology, and data interpretation	Steep learning curve; limited generalizability; variability in diagnostic performance	Establish structured training programs and certification systems; promote interdisciplinary collaboration
Data integration challenges	Functional endoscopy generates heterogeneous multimodal physiological data streams	Difficulty in comprehensive interpretation; fragmented diagnostic outputs	Develop integrated multimodal AI-assisted platforms capable of real-time data fusion and interpretation
Artificial intelligence transparency and ethics	AI-based classification systems may function as “black-box” decision models	Reduced clinician trust; medicolegal uncertainty; ethical concerns	Promote explainable AI models; establish regulatory and ethical frameworks for clinical deployment
Limited prospective clinical evidence	Current evidence remains largely proof-of-concept and technology-driven	Uncertain clinical utility and adoption barriers	Conduct large-scale multicenter prospective trials evaluating diagnostic accuracy, therapeutic impact, and patient outcomes
Translation from laboratory innovation to routine practice	Many technologies remain confined to specialized research settings	Delayed clinical implementation and limited accessibility	Encourage translational collaboration among engineers, gastroenterologists, AI scientists, and industry partners
Integration into precision medicine pathways	Lack of established theranostic workflows linking diagnosis to individualized therapy	Continued reliance on empirical treatment strategies	Develop companion diagnostic paradigms integrating physiological phenotyping with targeted therapeutic decision-making

AI, artificial intelligence.

### 4.1 Time and cost efficiency

A critical challenge should be directly confronted: would integrating complex physiological assessment modules into an already saturated endoscopic workflow substantially prolong procedure times and strain limited healthcare resources? This concern is not unfounded. From a dialectical perspective, however, the targeted assessment strategy advocated by functional endoscopy may paradoxically generate efficiency gains, although this hypothesis remains unproven and requires prospective workflow evaluation studies. Currently, random mucosal biopsies performed to exclude occult microscopic inflammation yield exceedingly low diagnostic returns, yet consume substantial pathology processing time and labor (42). If techniques such as confocal laser endomicroscopy or endocytoscopy could identify regions of barrier disruption or micro-inflammation in real time during the procedure, a shift from random to targeted biopsy could be achieved (11). This transition would increase diagnostic information density per procedure while reducing unnecessary biopsies, thereby optimizing time and cost allocation across the entire workflow. However, direct evidence of net workflow efficiency gains remains limited. Future studies should formally assess procedure duration, pathology workload, diagnostic yield, and cost-effectiveness using prospective implementation models before routine adoption can be justified.

### 4.2 Absence of standardization and consensus

The multidimensional physiological data generated by functional endoscopy—ranging from mucosal impedance values and fluorescein leakage areas to mast cell counts and pressure–volume curves—currently lack unified interpretative standards comparable to the Chicago Classification in esophageal motility (43). This gap constitutes a critical bottleneck impeding clinical adoption. More concerningly, premature dissemination of these technologies without clinical utility evidence and standardized interpretative criteria risks provoking technology-driven rather than medicine-driven overdiagnosis (44). In such a scenario, incidental or borderline physiological deviations may be erroneously pathologized. Establishing multicenter prospective clinical cohorts, defining normal reference ranges for each parameter, and reaching disciplinary consensus on reporting standards therefore represent foundational tasks that demand urgent attention.

### 4.3 Patient acceptability

The clinical adoption of any novel technology ultimately hinges on patient acceptability, where two specific concerns warrant attention. The first is the confounding effect of sedation depth. Accurate assessment of motility parameters requires a degree of preserved consciousness and muscular tone, which stands in direct tension with the deep propofol sedation now predominant in gastrointestinal endoscopy (45). Potential approaches to resolving this sedation–physiology conflict include conscious sedation protocols, ultrathin transnasal endoscopy, and emerging minimally sedating procedural strategies; however, comparative evidence substantiating their relative efficacy remains limited (46–48). Reconciling patient comfort with the preservation of physiological authenticity presents a challenge requiring nuanced calibration, and optimizing physiological assessment conditions should consequently be regarded as a priority research agenda rather than a solved technical problem. The second concern is patient tolerance for prolonged procedures. Adding balloon distension protocols, perfusion tests, and other functional assessment modules inevitably extends examination time, posing physiological and psychological demands on the patient (49). Modular, selectively combinable functional assessment workflows, coupled with thorough pre-procedural informed communication, will therefore be critical strategies for enhancing clinical acceptability. The stepwise resolution of these challenges will determine whether functional endoscopy moves from concept to clinical reality or remains confined within the pages of academic speculation.

### 4.4 Translational roadmap for functional endoscopy

**Figure 2:**
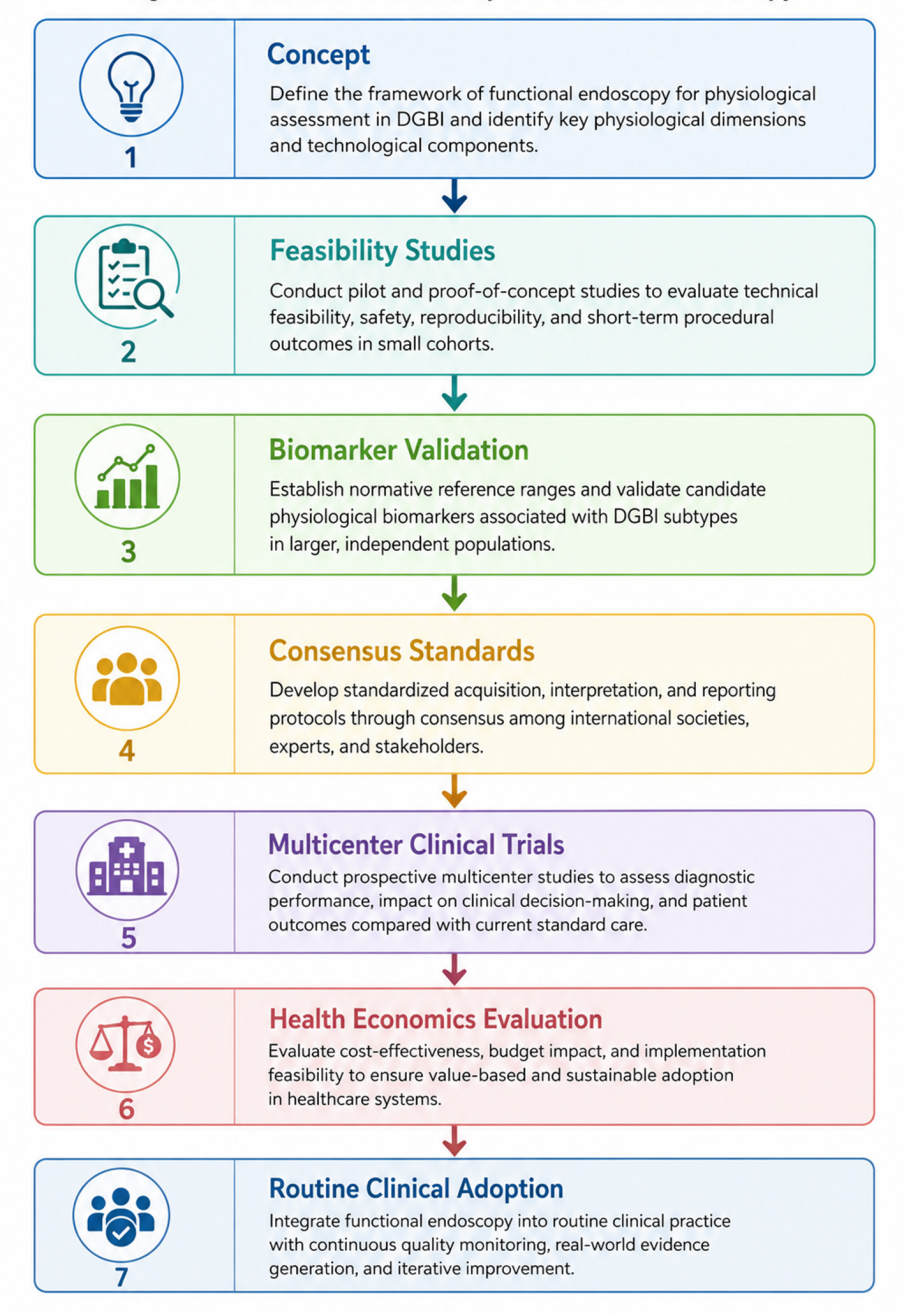
Translational roadmap of functional endoscopy.

The transition from a conceptual framework to routine clinical application will require a staged implementation strategy (Figure 2). First, multicentre observational studies should establish normative reference ranges and define reproducible physiological biomarkers (50). Second, international consensus groups—including the Rome Foundation, major gastrointestinal endoscopy societies, and neurogastroenterology organizations—should develop standardized acquisition and reporting protocols (51). Third, prospective outcome-based studies should evaluate whether physiology-guided phenotyping improves diagnostic accuracy, therapeutic selection, and patient outcomes compared with existing symptom-based algorithms. Finally, health-economic analyses and implementation science studies will be necessary before widespread clinical adoption can be recommended.

## 5 Toward precision endoscopic phenotyping based on physiological mechanisms

The technological framework and critical analysis presented above converge on a long-term translational objective. Importantly, the concepts discussed in this section should be viewed as future research directions and hypothesis-generating possibilities rather than immediately implementable clinical strategies. This section outlines this future blueprint across three dimensions: diagnostic reporting paradigms, artificial intelligence empowerment, and intrinsic therapeutic coupling.

### 5.1 Redefining the “negative endoscopy”

The statement “endoscopy reveals no abnormalities” has long since demonstrated its clinical inadequacy in the context of functional gastrointestinal disorders (4, 52). We advocate for a fundamental reformulation of the endoscopic reporting paradigm: descriptive physiological assessment should replace simple exclusionary statements. The ideal post-procedure report should not end with “mucosa appears smooth, no erosions seen.” Instead, it should integrate multidimensional physiological parameters acquired during the procedure, yielding a comprehensive interpretation with phenotyping significance. Diagnostic formulations such as “irritable bowel syndrome with endoscopically demonstrated duodenal barrier impairment” or “functional dyspepsia with evidence of visceral hypersensitivity” would directly anchor subjective symptoms to objective intraluminal physiological phenotypes (53, 54). This shift represents far more than a semantic update; it constitutes a pivotal step in moving the diagnosis of functional gastrointestinal disorders from symptom clustering toward mechanism-based phenotyping.

### 5.2 Artificial intelligence empowerment

Current artificial intelligence research in gastrointestinal endoscopy focuses heavily on polyp detection and classification (55, 56). Yet the data generated by functional endoscopy extend far beyond macroscopic morphology. We posit that the truly transformative value of artificial intelligence in this domain lies in the fusion modeling of multimodal physiological data. Specifically, feeding multidimensional parameters—mucosal impedance values, spatiotemporal patterns of smooth muscle motor activity, eosinophil and mast cell density and activation status, and spectral fingerprints of intraluminal metabolites—into deep learning networks could enable unsupervised subtype discovery and individualized prediction (57, 58). Such models hold the potential to uncover complex physiological associations imperceptible to both the human eye and conventional statistical methods, thereby constructing a genuinely mechanism-driven classification system. Although multimodal artificial intelligence models may eventually facilitate such mechanism-oriented phenotyping, several critical obstacles currently limit progress (59). Most importantly, no accepted physiological ground truth exists for DGBI subtypes, which makes supervised model development inherently challenging (60). Therefore, future research should prioritize the establishment of large-scale multimodal physiological datasets, the development of consensus phenotype definitions, and the advancement of explainable artificial intelligence frameworks before clinical deployment can be realistically considered.

### 5.3 Intrinsic therapeutic coupling

The ultimate value of functional endoscopy extends beyond diagnosis to direct therapeutic coupling. One disruptively promising direction is real-time *in vivo* pharmacodynamic assessment under endoscopic guidance. Local perfusion of histamine antagonists, mast cell stabilizers, or specific receptor blockers to the target mucosa via the biopsy channel, combined with real-time observation of dynamic changes in barrier function indices or sensory nerve activation markers, could enable *in vivo* drug sensitivity screening within a single endoscopic session (61, 62). This “companion diagnostics” paradigm transforms the endoscope from a static observational window into a dynamic therapeutic decision-making platform. It shifts the treatment of functional gastrointestinal disorders from empirical trial-and-error toward precision intervention grounded in individual endoscopic phenotypes (63). Collectively, these three trajectories illustrate a potential future pathway through which functional endoscopy may evolve from a diagnostic instrument into a more integrated theranostic platform. However, substantial technological, biological, and clinical validation will be required before such applications can be realized.

## 6 Summary

This perspective article proposes a conceptual framework for rethinking the role of gastrointestinal endoscopy in DGBI. We emphasize that functional endoscopy should currently be regarded as a translational research agenda rather than an established clinical standard. Its ultimate value will depend on future evidence demonstrating reproducibility, clinical utility, cost-effectiveness, and patient benefit. Although many components of this framework remain at varying stages of technological and clinical maturity, the concept offers a potential roadmap for future mechanism-based endoscopic assessment and warrants systematic prospective validation. It should be reshaped into an “intraluminal decoder” capable of penetrating the facade of “normal mucosa” and deciphering the complex pathophysiological mechanisms underlying these disorders. This paradigm shift captures real-time signals across motility, barrier function, visceral perception, and immune activation. Realizing this vision depends on the synergistic advancement of optical biopsy, functional sensing, and molecular imaging technologies; on the fusion modeling of multimodal data through artificial intelligence; and, crucially, on the systemic reconfiguration of reporting paradigms and clinical pathways.

We call upon the gastroenterology community to transcend the constraints of conventional morphological thinking, to actively engage with the translational potential of intraluminal functional assessment technologies, and to validate the clinical utility and incremental value of functional endoscopic phenotyping through multicenter prospective studies. Only through such concerted efforts can a new era be inaugurated for the hundreds of millions of patients suffering from functional gastrointestinal disorders—an era that moves decisively from empirical symptom management toward mechanism-driven precision diagnosis and therapy.

## Data Availability

The original contributions presented in the study are included in the article/supplementary material, further inquiries can be directed to the corresponding author.
